# Case report: Successful simultaneous heart-kidney transplantation across a positive complement-dependent cytotoxic crossmatch

**DOI:** 10.3389/fneph.2022.1047217

**Published:** 2022-11-28

**Authors:** Takayuki Yamamoto, Daniel S. Pearson, Emad I. Ababneh, Cynthia Harris, Pitchaphon Nissaisorakarn, Grace K. Mahowald, Yael K. Heher, Nahel Elias, James F. Markmann, Gregory D. Lewis, Leonardo V. Riella

**Affiliations:** ^1^ Center for Transplantation Sciences, Massachusetts General Hospital/Harvard Medical School, Boston, MA, United States; ^2^ Department of Surgery, Massachusetts General Hospital/Harvard Medical School, Boston, MA, United States; ^3^ Histocompatibility Laboratory, Department of Pathology, Massachusetts General Hospital, Boston, MA, United States; ^4^ Department of Pathology, Massachusetts General Hospital, Boston, MA, United States; ^5^ Division of Nephrology, Massachusetts General Hospital/Harvard Medical School, Boston, MA, United States

**Keywords:** heart transplant, kidney transplant, highly sensitized, CDC positive, eculizumab, DSA positive

## Abstract

Preformed donor-specific antibodies are associated with a higher risk of rejection and worse graft survival in organ transplantation. However, in heart transplantation, the risk and benefit balance between high mortality on the waiting list and graft survival may allow the acceptance of higher immunologic risk donors in broadly sensitized recipients. Transplanting donor-recipient pairs with a positive complement dependent cytotoxic (CDC) crossmatch carries the highest risk of hyperacute rejection and immediate graft loss and is usually avoided in kidney transplantation. Herein we report the first successful simultaneous heart-kidney transplant with a T- and B-cell CDC crossmatch positive donor using a combination of rituximab, intravenous immunoglobulin, plasmapheresis, bortezomib and rabbit anti-thymocyte globulin induction followed by eculizumab therapy for two months post-transplant. In the year following transplantation, both allografts maintained stable graft function (all echocardiographic left ventricular ejection fractions ≥ 65%, eGFR>60) and showed no histologic evidence of antibody-mediated rejection. In addition, the patient has not developed any severe infections including cytomegalovirus or BK virus infection. In conclusion, a multitarget immunosuppressive regimen can allow for combined heart/kidney transplantation across positive CDC crossmatches without evidence of antibody-mediated rejection or significant infection. Longer follow-up will be needed to further support this conclusion.

## Introduction

Highly sensitized patients present a major challenge in transplantation due to the long waiting times required to find a compatible organ donor. The presence of donor specific antibodies (DSA), especially in the context of a positive T- and B- cell complement-dependent cytotoxic (CDC) crossmatch, is associated with a high risk of acute antibody-mediated rejection (AMR) and poor graft survival (1–5). In kidney transplantation, a positive T-cell CDC crossmatch is considered a contraindication to transplant. However, kidney transplant candidates can be on dialysis while awaiting a compatible kidney, while heart transplant candidates must rely on more limited strategies to bridge them to transplant. Furthermore, strategies to reduce anti-HLA antibodies in highly sensitized patients have limited efficacy (6). The peri- and post-transplant use of the complement inhibitor eculizumab represents a potential approach that has shown some promise in reducing the risk of developing acute AMR in highly sensitized patients receiving heart (7) and kidney transplants (8, 9). Despite these advances, performing heart or kidney transplants with known DSA and positive T- and B-cell CDC crossmatches remain controversial due to a high risk of graft loss (1, 2, 10).

Here we present the first case report, to the best of our knowledge, of a successful simultaneous heart and kidney transplant (SHK) in a highly sensitized recipient across significant DSA and positive CDC crossmatches using the complement inhibitor eculizumab to augment standard induction therapy.

## Case presentation

**Figure 1:**
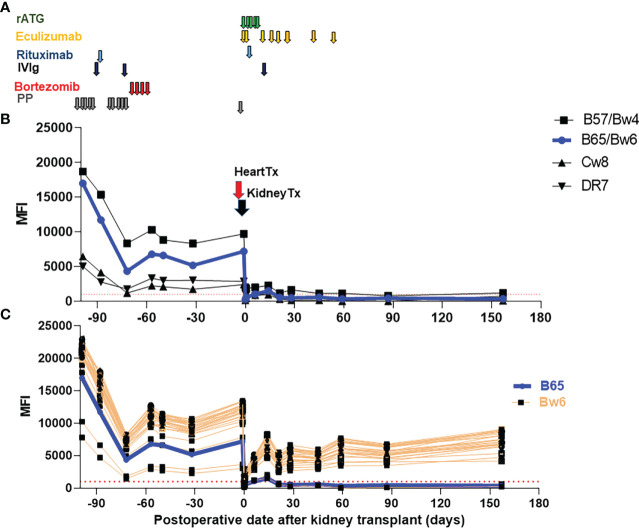
Immunosuppressive therapies and trend of donor specific antibodies (DSA). **(A)** Immunosuppressive therapies used pre-transplant and early post-transplant. Maintenance therapies are not depicted (tacrolimus, mycophenolate mofetil and a steroid taper.) **(B)** Class I and II DSA following desensitization and SHK transplantation. **(C)** Reactivity of B locus antigens containing the shared epitope Bw6. B65/Bw6 (DSA) reactivity is shown in blue while reactivity against all other Bw6 containing antigens is shown in orange. The threshold of positive reactivity for both class I and II antibodies is ≥1000 MFI (red dotted lines). rATG, rabbit anti-thymoglobulin, IVIg, intravenous immunoglobulin, PP, plasmapheresis, Tx, Transplant.

A 40-year-old female with a past medical history of a congenital membranous ventricular septal defect and cocaine use disorder in remission presented to our institution in January 2020 with an inferior ST segment elevation myocardial infarction with a high sensitivity Troponin T in excess of 40,000 ng/L. Her course was complicated by papillary muscle rupture, severe mitral regurgitation, cardiogenic shock and acute kidney injury. She was briefly on veno-arterial extracorporeal membrane oxygenation and continuous veno-venous hemofiltration but partially recovered renal function after emergent intervention which included bioprosthetic mitral valve replacement. In the following months she had persistent heart failure and declining renal function. She had chronic kidney disease for more than 2 years with estimated glomerular filtration rate below 30 ml/min, which progressed slowly up to her need to initiate dialysis close to the time of transplant. Of note, the patient had a history of >10 RBC transfusions and two pregnancies. She was listed as a candidate for heart and kidney transplant and admitted for desensitization in the setting of a high cPRA (calculated panel reactive antibodies by means of Luminex solid phase testing; combined: 99%, class I: 98%, class II: 60%). Desensitization therapy comprised of 10 sessions of plasmapheresis (PP), 1g rituximab, and 2 doses of 1g/kg intravenous immunoglobulin (IVIg) followed by 1 cycle of bortezomib (1.3 mg/m^2^, days 1, 4, 8, and 11) from day-97 to -57 (**Figure 1A**). Post-desensitization anti-HLA antibody titers were reduced by approximately 60-75% (**Figure 1B**). However, her cPRA did not change significantly as most antibody reactivities did not decrease below our threshold for positivity (mean fluorescence intensity (MFI) ≥1000).

Eventually, the patient received a deceased brain dead (DBD) donor organ offer to which numerous DSA were present (B57, B65/Bw6, Cw8, DR7) with the most recent serum available to the HLA laboratory (which was approximately 1 month after desensitization was completed and 1 month prior to transplant). Using a sample drawn at the time of the heart transplant, the flow cytometric and CDC crossmatches were positive for both T- and B-cells. Both flow crossmatches were strongly positive; Flow T cell delta fluorescence units (DFU) was 9,002 (positive is >=150 DFU) while flow B cell DFU was 14,772 DFU (positive is >=3000 DFU).

Additionally, the patient also appeared to have DSA to Bw6, an antigen shared with many B locus antigens (11), with an average MFI ~10,000 (**Figure 1C**). This was particularly concerning as even low level anti-Bw6 antibodies can lead to allograft rejection (12). However, due to the patient’s rapidly declining clinical status, the offer was accepted and heart followed by kidney transplantation following the DUET protocol was performed (7). Briefly, 1200 mg of eculizumab was given at time of heart transplant and 900 mg at the time of the kidney transplant. Thereafter, eculizumab was continued 900 mg weekly until day 56 (**Figure 1A**). For induction therapy, rabbit anti-thymocyte globulin (ATG) (1.5mg/kg x 4 doses on day 0-3 + 1mg/kg on day 4), 1g of rituximab on day 1, and 1g/kg of IVIg on day 7 were used. Maintenance therapy included tacrolimus, mycophenolate mofetil (MMF) and a steroid taper.

Post-transplant, her heart allograft function was stable with a consistently robust left ventricular ejection fraction (EF) in excess of 65% and normal cardiac filling pressures and cardiac indices on right heart catheterizations. She required vasopressors initially post-transplant that were completely weaned by day 12. Over a 4-week period spanning post-operative days 35-63, heart biopsies showed low-level grade 1A/1R to 2A/1R acute cellar rejection (ACR) despite no evidence of AMR. No additional anti-rejection therapy such as steroid pulse or ATG was required. Outside of this self-limited period of time, there has been no evidence of cellular rejection (all biopsies ≤ 1A/1R). Left ventricular function has remained within the normal range post-transplant with all LVEFs >65% over 1 year after the transplant.

**Figure 2:**
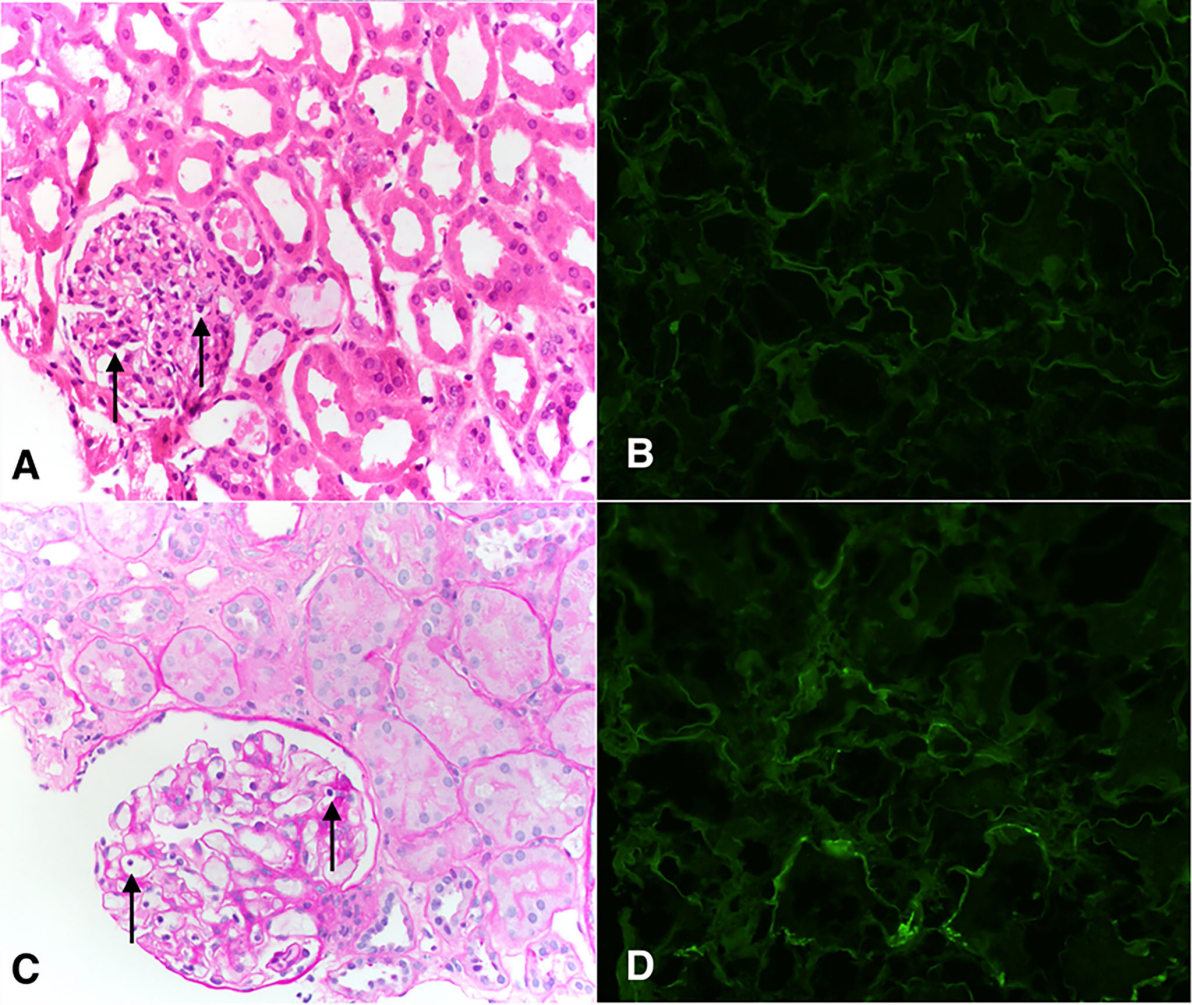
Pathological findings of the transplanted kidney on days 8 **(A, B)** and 380 **(C, D)**. **(A)** Representative H&E staining showing glomeruli with focal presence intracapillary neutrophils and lymphocytes, compatible with Banff glomerulitis g1 score (arrows). Adjacent tubules show attenuation and loss of brush borders typical of acute tubular injury. Peritubular capillaries are not dilated and do not show increased circulating inflammatory cells. Interstitial inflammation is minimal. **(B)** Peritubular capillaries are negative for C4d by immunofluorescence (c4d0). **(C)** Glomerulus shows intracapillary lymphocytes (arrows). This was present in <25% of glomeruli (g1). Interstitial inflammation is minimal (i0), there is no tubulitis (t0), and there is no significant peritubular capillaritis. **(D)** Peritubular capillaries show predominantly negative C4d staining by immunofluorescence (c4d0).

From the kidney perspective, the patient had delayed graft function and required renal replacement therapy (RRT) temporarily post-transplant. Kidney allograft biopsies performed on day 8 and day 15 showed acute tubular injury and mild glomerulitis (only on day 8) but no rejection [day 8: t0, i0, v0, g1, ptc0, C4d: neg, cg0, ah0, ci0, ct0, cv0, ti0, mm0 (**Figures 2A, B**) and day 15: t0, i0, v0, g0, ptc0, C4d: neg, cg0, ah0, ci0, ct0, cv0, ti0, mm0]. By day 47, renal function had recovered and RRT was discontinued. Subsequently, her kidney function has been stable (creatinine 1 mg/dL mg/dL at 15 months post-transplant without any proteinuria) and repeat allograft kidney biopsies performed on day 324 and 380 showed only mild microvascular inflammation and IF/TA grade I [day 324: t0, i0, v0, g1, ptc0, C4d:neg, cg0, ah0, ci1, ct1, cv1,ti1, mm0 and day 380: t0, i0, v0, g1, ptc0, C4d:neg, cg0, ah0, ci0-1, ct0-1, cv0,ti0, mm0 (**Figures 2C, D**)].

Except for two episodes of urinary tract infections, the patient had no significant infectious complications such as CMV, EBV, or BKV viremia and no significant adverse events related to the desensitization protocol such as cytopenia, GI side effects or polyneuropathy. Notably 6 months of valganciclovir was administered post-transplant in the setting of the donor organ being CMV IgG positive and the recipient CMV IgG negative at the time of transplant.

In regards to patient’s preformed DSA (B57, B65/Bw6, Cw8 and DR7), all precipitously dropped shortly after transplantation and stayed low in the subsequent follow-up period (**Figure 1B**). In contrast, reactivity to the vast majority of Bw6-containing antigens rebounded to approximately 50% of pre-transplant levels within weeks (**Figure 1C**).

## Discussion

Broad sensitization against HLA antigens represents a significant barrier to both kidney and heart transplantation. For example, highly sensitized candidates have significantly longer waiting times with an associated increase in waitlist mortality (3, 13, 14). While desensitization with PP and IVIG in living donor kidney transplant had some short- and mid-term success (1–5), it is still associated with significant risk for AMR and shortened graft survival. The majority of transplant centers currently avoid pre-formed DSA in patients with potential living donors by performing paired-kidney exchange. For deceased donor transplant candidates, finding a suitable donor without preformed DSA, in particular for those with cPRA>98%, remains a challenge even with changes in the kidney allocation policy that provide additional points depending on degree of sensitization.

**Figure 3:**
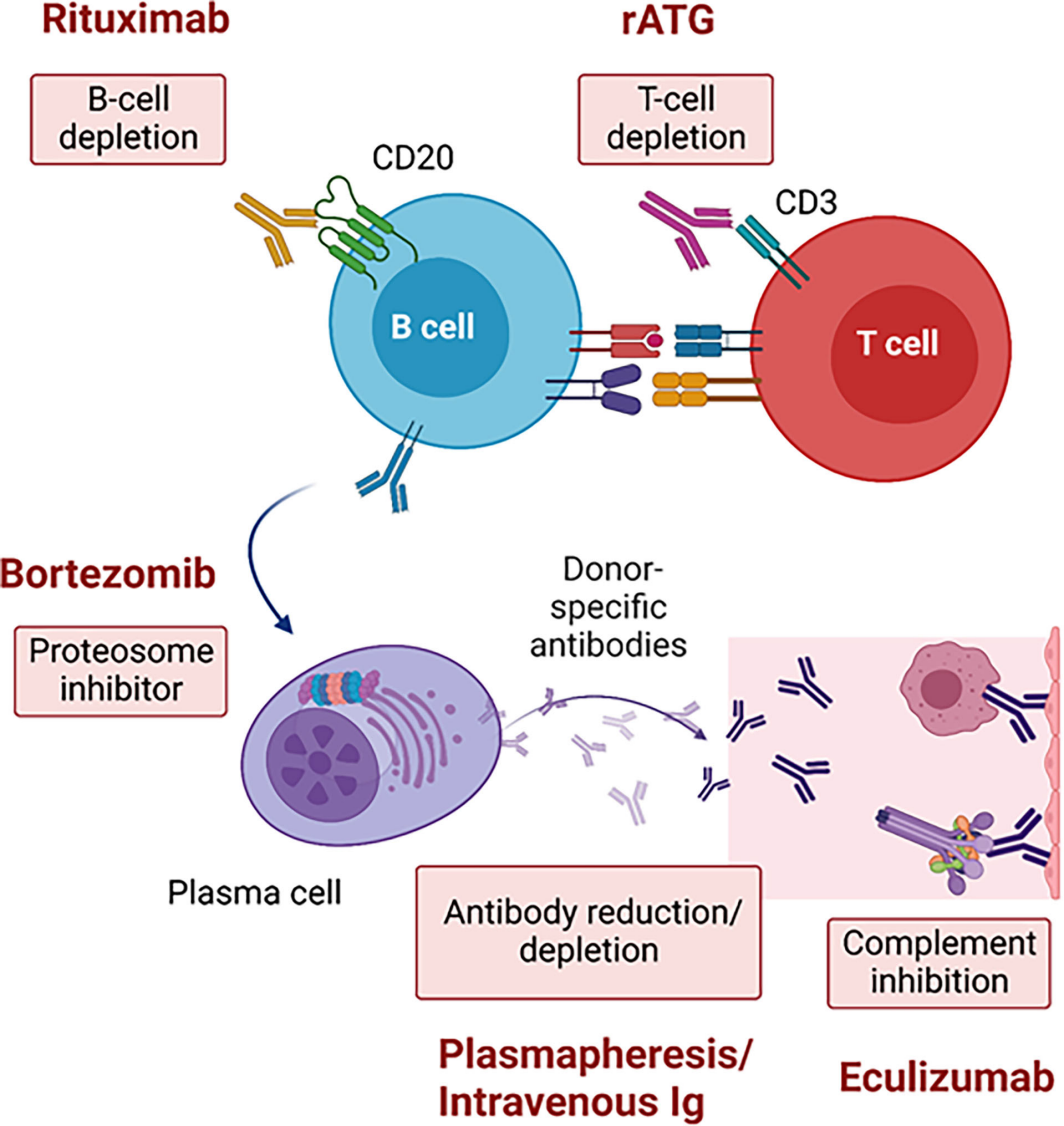
Strategies across a positive complement-dependent cytotoxic crossmatch due to donor-specific antibodies. rATG, rabbit anti-thymoglobulin.

Desensitization in heart transplantation has used similar approaches as in kidney transplantation with a combination of PP with IVIG and rituximab being the cornerstone. Success of those strategies have been limited (6) due to rapid rebound of antibodies. As a complementary strategy, complement inhibition post-transplant to reduce the risk of AMR in deceased donor kidney transplant recipients has been used with some success in the short-term by reducing the rate of AMR (9). Accumulating evidence supports the use of eculizumab to allow for heart transplantation (7, 13) with a positive CDC-crossmatch donor for selected candidates at high risk of death (15). However, kidney transplantation across a positive CDC-crossmatch is still avoided due to high risk of AMR and graft loss. There were several reports of successful CDC-crossmatch positive liver-kidney transplants (16, 17). However, the liver is immunologically distinct from other transplanted organs. It is well-known that transplanted livers contain large numbers of immune cells and through a variety of poorly understood mechanisms are more tolerogenic (18). Additionally, absorption of alloreactive antibodies by allograft livers can significantly reduce DSA in dual organ transplants if the liver is transplanted first (19). These phenomena have been exploited to confer immunological protection when livers are transplanted in combination with other solid organs (20) allowing for successful CDC-crossmatch positive liver-kidney transplants (21). However, there are no reports of a SHK with a CDC crossmatch positive donor. Given the declining clinical status of the patient presented, our team opted to proceed with pre-transplant therapy including combined IVIg/rituximab and bortezomib in addition to the DUET study protocol (ATG, rituximab and eculizumab) after transplant. Theoretically, this multimodal strategy would reduce T-cell dependent antibody production by depleting T cells with ATG, decrease naïve and memory B cells with rituximab, impair antibody production by plasma cells with bortezomib, neutralize and deplete circulating DSA with IVIg and PP, and block antibody function through the terminal complement inhibitor eculizumab (**Figure 3**).

After pre-transplant desensitization, the MFIs of the patient’s DSA were reduced relative to pre-desensitization (**Figure 1B**), though her cPRA remained unchanged. Given her high initial antibody titers, we hypothesized that desensitization in combination with standard induction plus eculizumab would decrease the patient’s risk of acute AMR. We observed that DSA MFIs dropped substantially immediately after heart-kidney transplants, which could at least be partly attributed to antibody adsorption of graft’s endothelial cells (22).

Except for the reactivity against the shared epitope Bw6 (MFIs ~5000-7000) (**Figure 1C**), MFIs to DSA remained low post-transplant (class I: MFI<2000, class II: MFI <1000) (**Figure 1B**), which is a favorable sign associated with lower risk of AMR. The pattern of HLA antibody reactivity we observed pre-transplant was consistent with an anti-Bw6 antibody. This was particularly concerning since reactivity to broadly-shared epitopes can underestimate the true strength of DSA due to spreading of the antibody across multiple beads in single antigen bead assays (23). Surprisingly, there was no clinical or histologic evidence of AMR in the setting of this rapid and sustained increase in anti-Bw6 reactivity. However, discerning true reactivity against the Bw6 epitope from reactivity to combinations of other shared epitopes can be difficult (24). Post-transplant, the reactivity to B65/Bw6 (DSA) diverged from reactivity to other Bw6 antigens (non-DSA) (**Figure 1C**), indicating that the patient’s antibody was likely not a true Bw6 antibody.

Treatment with rituximab and bortezomib likely suppresses DSA titers through the reduction of CD20+/CD27+ memory B cells in the blood and bone marrows as well as impairs DSA production by short and long-lived plasma cells by proteosome inhibition (25). This may result in hypogammaglobulinemia which increases the risk of infection. However, the addition of IVIg therapy likely mitigated that risk. In addition, mature B cells recover more rapidly returning to baseline by six months, whereas memory B cells remain low at two years after rituximab therapy. Together, this may explain why there were no significant infectious complications post-transplant.

A limitation of study is that we report just 17 months follow-up after SHK. While the patient has demonstrated no evidence of AMR to-date, longer follow-up will be required to evaluate the long-term impact of this high immunologic risk transplantation.

## Conclusion

Desensitization with IVIg/PP/rituximab/bortezomib followed by ATG and a 2-month eculizumab course allowed for the successful SHK across positive T- and B- cell CDC crossmatches. One- year post-transplant, there were no significant adverse events including AMR and infectious complications. Our strategy may be a promising approach to widen the potential donor pool to include CDC crossmatch positive donors for patients with significant DSA who are refractory to desensitization and have a higher mortality on the waiting list.

## Data availability statement

The original contributions presented in the study are included in the article/Supplementary Materials, further inquiries can be directed to the corresponding author/s.

## Ethics statement

Ethical review and approval was not required for the study on human participants in accordance with the local legislation and institutional requirements. The patients/participants provided their written informed consent to participate in this study. Written informed consent was obtained from the individual(s) for the publication of any potentially identifiable images or data included in this article.

## Author contributions

TY and LR designed the case report. TY, DP, EA and LR wrote the article. TY, DP, EA, CH, PN, GM, YH, NE, JM, GL, and LR involved the case report. All authors contributed to the article and approved the submitted version.

## Funding

This work was in part supported by the following research grant from the National Institutes of Health (NIH) Grant R01 AI143887 (to LR).

## Conflict of interest

The authors declare that the research was conducted in the absence of any commercial or financial relationships that could be construed as a potential conflict of interest.

## Publisher’s note

All claims expressed in this article are solely those of the authors and do not necessarily represent those of their affiliated organizations, or those of the publisher, the editors and the reviewers. Any product that may be evaluated in this article, or claim that may be made by its manufacturer, is not guaranteed or endorsed by the publisher.
